# Late Life Employment Histories and Their Association With Work and Family Formation During Adulthood: A Sequence Analysis Based on ELSA

**DOI:** 10.1093/geronb/gbx066

**Published:** 2017-05-31

**Authors:** Morten Wahrendorf, Paola Zaninotto, Hanno Hoven, Jenny Head, Ewan Carr

**Affiliations:** 1 Centre for Health and Society, Institute for Medical Sociology, Medical Faculty, University of Düsseldorf, Germany; 2 Department of Epidemiology and Public Health, University College London, UK

**Keywords:** Extended working life, England, ELSA, Life course conditions, Sequence analysis

## Abstract

**Objectives:**

To extend research on workforce participation beyond age 50 by describing entire employment histories in later life and testing their links to prior life course conditions.

**Methods:**

We use data from the English Longitudinal Study of Ageing, with retrospective information on employment histories between age 50 and 70 for 1,103 men and 1,195 women (*n* = 2,298). We apply sequence analysis and group respondents into eight clusters with similar histories. Using multinomial regressions, we then test their links to labor market participation, partnership, and parenthood histories during early (age 20–34) and mid-adulthood (age 35–49).

**Results:**

Three clusters include histories dominated by full-time employees but with varying age of retirement (before, at, and after age 60). One cluster is dominated by self-employment with comparatively later retirement. Remaining clusters include part-time work (retirement around age 60 or no retirement), continuous domestic work (mostly women), or other forms of nonemployment. Those who had strong attachments to the labor market during adulthood are more likely to have histories of full-time work up until and beyond age 60, especially men. Parenthood in early adulthood is related to later retirement (for men only). Continued domestic work was not linked to parenthood. Partnered women tend to work part-time or do domestic work. The findings remain consistent after adjusting for birth cohort, childhood adversity, life course health, and occupational position.

**Discussion:**

Policies aimed at increasing the proportion of older workers not only need to address later stages of the life course but also early and mid-adulthood.

In response to demographic ageing, there is an increasing interest in understanding the determinants of labor market participation beyond age 50. This has led to numerous studies investigating retirement behavior (see Fisher, Chaffee, & Sonnega, 2016 for a recent review). Results of this research aid in identifying factors related to retirement behavior, and thus, help to develop measures to promote extended working lives. However, most studies are based on occupational cohorts, often recruited during midlife. In these studies, characteristics in midlife (e.g., working conditions) are usually linked to the likelihood of retirement during a follow-up period. This focus on midlife conditions and transitions into retirement has, however, at least two important consequences for existing knowledge.

A first shortcoming relates to the measurement of retirement, or more generally, the measurement of labor market participation at older ages. Most studies focus on transitions into retirement and reduce the complexity of labor market participation to a single outcome (retirement timing) without studying entire trajectories or patterns of later life employment histories. This neither considers how retirement behavior is embedded within larger histories, nor—more generally—does it recognize various types of employment patterns (Sackmann & Wingens, 2003). To describe employment histories in later life, for example, not only the age by which workers retire is important, but the situation from which he or she retires is also part of the history. More specifically, whether the person was employed or self-employed before retiring, or whether they previously worked part or full-time are important factors (Blanchflower, 2000; McNair, Flynn, Owen, Humphreys, & Woodfield, 2004; Parker & Rougier, 2007). In other words, a more comprehensive approach is needed to describe complete patterns of labor market participation at older ages; one, where retirement is not isolated from larger histories but where various forms of labor market involvements covering an extended time frame are considered (Aisenbrey & Fasang, 2010). Notably, this broader perspective does not require that people retire in the study period or work at study onset, thus, correcting for a possible gender bias of previous research where women are underrepresented (Worts, Corna, Sacker, McMunn, & McDonough, 2016).

A second shortcoming of existing research is that little is known about links between later labor market participation (beyond age 50) and conditions earlier in the life course, such as childbearing, work, and partner histories. Specifically, while parenthood and childbearing responsibilities were linked with lower workforce participation among women during adulthood (see Baranowska-Rataj & Matysiak, 2016 for a review), the question of whether this holds true for employment participation beyond age 50 is less explored. For example, using European data from the Survey of Health Ageing and Retirement in Europe (SHARE), having a higher number of children was linked with later retirement for men (Hank & Korbmacher, 2013). Yet, no such association was found for women (across 13 continental European countries). Or, another study from Australia suggests that women with caring responsibilities during adulthood have more difficulties in developing a continuous working career than men, possibly due to cultural expectations and existing gender roles (Majeed, Forder, Mishra, Kendig, & Byles, 2015). However, a study from Britain found that women with a higher number of children (between ages 20 and 60) were more likely to work after age 60 (Finch, 2014). In that case, the explanation could be that women need to compensate for their lower pension contributions during adulthood, and therefore, work longer in later life. The latter finding is supported by studies that investigate the links between labor market attachments during adulthood and later life labor market participation. For example, based on the Swiss subsample of SHARE (Madero-Cabib, Gauthier, & Le Goff, 2016), both men and women with weak ties to the labor market during adulthood were more likely to retire late (after the state pension age). Similar results come from three American studies (Cahill, Giandrea, & Quinn, 2012; Clarke, Marshall, & Weir, 2012; Raymo, Warren, Sweeney, Hauser, & Ho, 2011), where men and women with unstable careers, who were self-employed, or worked in jobs that had no retirement plans were more likely to have extended working lives. Yet, others studies find that those who already had strong ties to the labor market also continue to work at older ages (Pienta, Burr, & Mutchler, 1994). Turning to marital histories, findings generally suggest that married women are more likely to retire earlier compared to unmarried women (but not men) (Finch, 2014; Madero-Cabib et al., 2016) and that being single is related to later retirement (both for men and women). Taken together, there is some evidence that life course conditions, and in particular childbearing responsibilities and labour market participation, are linked with later life employment histories. But findings also show that there are important gender-differences and that the country context, as well as existing pension contributions, all matter (Hank & Korbmacher, 2013; Worts et al., 2016).

In sum, despite a growing body of research on predictors of retirement, few have investigated complete patterns of labor market participation at older ages and their association with work and family formation during adulthood. From a policy perspective, such studies would help to identify entry points for policy measures aiming to extend working lives, and provide an in-depth description of older people’s labor market participation. Along these lines, using life history data from the English Longitudinal Study of Ageing this paper has two aims: First, we use sequence analyses to summarize complete employment histories of men and women in later life from the ages 50–70 (Abbott, 1995; Aisenbrey & Fasang, 2010). As a second aim, the study investigates how types of later life employment histories relate to circumstances during early and mid-adulthood, including labor market participation, partnership status, and parenthood. In both aims, we examine if there are gender differences, that is, if histories and their associations to life course conditions differ for men and women.

Extending current research along these two aims is concordant with the “life course perspective” as the predominant framework of sociological and epidemiological research of today (Elder, Johnson, & Crosnoe, 2003; Kuh, Ben-Shlomo, Lynch, Hallqvist, & Power, 2003). Therefore, the following paragraph briefly describes our broader theoretical perspective.

## The Life Course Perspective

To advance the study of late life employment patterns, researchers increasingly argue that research needs to adopt a life course perspective. This not only means that research should study long-term effects based on longitudinal data, but also, that labor market participation is best understood when specific principles that shape individual life courses are considered (Elder et al., 2003; Kuh et al., 2003; Sackmann & Wingens, 2003). Among these principles, one highlight that research needs to take a long view of biographies, covering an extended time frame (George, 2013). Specifically, this refers to the above-mentioned idea that life course research should not only examine the timing of specific “transitions” (e.g., from paid work into retirement) but should also take a more holistic perspective that describes entire patterns of life course “trajectories” (Aisenbrey & Fasang, 2010; Sackmann & Wingens, 2003; Worts et al., 2016). A crucial development, in this respect, is the growing popularity of sequence analysis (Abbott, 1995; Aisenbrey & Fasang, 2010; Studer & Ritschard, 2016), which enables the development of a typology of life course trajectories. The first aim of our study, therefore, is to identify types of late life histories.

The life course perspective also recognizes that individual lives, including employment histories, are best understood within the context of previous experiences and those made in other domains, such as partnership and family histories (Han & Moen, 1999). This invokes the concept of cumulative advantages or disadvantages throughout the life course (Dannefer, 2003). This concept refers to the idea that disadvantages accumulate across the life course, suggesting that those who were excluded from the labor market during adulthood may have greater difficulties finding a job later in life. Therefore, the second aim of our study is to link types of late life employment histories to work and family formation during adulthood.

Furthermore, the life course perspective underlines that biographies are best understood in the light of their institutional and cultural contexts in which they unfold (for our sample in England and Wales between the 1980s and early 2000s). In case of employment histories, this includes the extent to which public and private pensions are available and the age at which a person is usually eligible for a full pension. Although our analyses do not aim to compare different countries or cohorts, such contextual framing enables a clearer interpretation. In our sample of adults living in England and Wales, the state pension age is 60 for women and 65 for men, while the level of public state pension is low compared with other European countries. Alongside the state pension system, the majority of employees will belong to a workplace pension scheme, in particular men (Banks, Emmerson, & Blundell, 2005). In addition, traditional gender roles mean that women spend more time doing housework than men (Kan, 2008). As such, with regard to the first aim of our study, we are likely to find that many people continue working until state pension age, but also that women are more likely to be involved in part-time paid work or housework compared with men.

In sum, the paper has two research questions: (a) Which types of late life employment histories can be distinguished among older men and women? (b) How are circumstances during early and mid-adulthood related to late life employment histories for men and women?

## Methods

### Data Source

Data are drawn from the English Longitudinal Study of Ageing (ELSA), a nationally representative survey of people aged 50+ living in private households in England (Steptoe, Breeze, Banks, & Nazroo, 2013). Data collection has taken place biennially since 2002, consisting of face-to-face interviews and self-completion questionnaires. Our analyses draw upon information from the third wave of ELSA (2006–2007) (Scholes et al., 2009), which included continuing respondents (core members who joined ELSA at study onset) as well as new members from younger cohorts (born after 1952) who were added at wave three to maintain age representation (so called “refreshers’’). In addition to the “regular” interview focusing on current circumstances, the 2006–2007 round of ELSA included a separate retrospective interview on previous life course conditions and employment histories. To improve the quality of retrospective information given by the respondent, ELSA collected life history data using “calendar interviews,” where recall and timing of information is supported by a graphical representation that is filled out during the interview (Belli, 1998). More specifically, a calendar with different life domains (e.g., work, partnership, and children) supports respondents in remembering their prior life courses. This approach was first developed as a self-completion questionnaire (Blane, 1996) and subsequently transformed into a computer-assisted personal interview by the U.K. National Centre for Social Research (Scholes et al., 2009). In contrast to conventional methods of data collection, studies show that calendar interviews improve the accuracy of retrospective information (Belli, Smith, Andreski, & Agrawal, 2007; Drasch & Matthes, 2011). Furthermore, the life grid approach allows for comparable information (referring to different time points) to be collected, without producing missing data due to panel attrition in a prospective survey. Also, calendar interviews ensure that illogical sequences are not reported.

The third wave of ELSA includes life history data from 7,855 men and women (with an individual response rate of 73% among core members). For our study, we investigate late life employment histories from ages 50–70. The lower age limit was set to 50 because earlier stages did not appear relevant for the study of later life histories, and because including earlier stages would have increased the number of potential histories. The upper age limit of 70, in turn, not only allows us to study early exits from the labor market (before age 60) and workforce participation up to public state pension age (60 for women and 65 for men in our sample) but it also covers potential labor market involvements beyond the state pension age. Furthermore, for those aged older than age 70, very few changes in labor market status were observed in our data. Since extending the upper limit of life histories (beyond age 70) would reduce the available sample (respondents must be at least as old as this upper limit), we therefore decided to set age 70 as the upper limit. As such, the analyses only include men and women aged 70 or older at time of the retrospective interview, and who have complete information on employment histories, consisting of 1,103 men and 1,195 women (*n* = 2,298).

### Measures

Two types of measure are at the core of our study: late life employment histories (between ages 50 and 70) and life course conditions.

#### Late life employment histories

ELSA collects details on respondents’ previous work history that was self-reported by the respondent within the calendar interview (see above). Information includes each job a respondent had during their working career (from age at first job till moment of the interview) and the following details: starting and ending date, whether the job was part-time or full-time and whether the respondent was an employee or self-employed worker. Where a respondent reports no paid job for a given period, the respondents was asked to describe the period, including whether it is due to retirement, domestic work, unemployment, or other possible states such as being sick or disabled, voluntary work, leisure activities, travelling or others. Only paid jobs lasting 6 months or longer and periods of nonemployment of 3 months or more are recorded. In this way, for persons aged 70 or older, we can derive their occupational situation at 21 time points (ages 50–70). From this, we distinguish seven situations (or “states”). In doing so, we aim at focusing and simplifying the measurement as far as possible, without losing the information of interest to describe late life employment histories. The seven states are: (a) “employed/full-time” (working 35 or more hours a week), (b) “employed / part-time” (working less than 35 hr a week), (c) “self-employed” (irrespective of working hours), (d) “unemployed,” (e) “domestic work” (looking after home or family), (f) “retired,” and (g) “not working” (all other forms of nonemployment). A number of other states could have been included. For example, we may have differentiated “not working” more explicitly but the importance of this distinction (and the prevalence of these states) appeared not relevant enough to warrant the additional complexity that would have been involved (the number of possible sequences grows extensively with numbers of states). In a small percentage of cases (4%), respondents report both a paid job and a period of nonemployment in the same year. This would be the case, for example, if the person stopped and started a new job in a year in which he also had a gap of unemployment. In this infrequent scenario, we decided to prioritize the information on the gap, because a break in a sequence is considered more important than the continuation of a spell.

In sum, our approach helps to account for different forms of labor market situation and to describe late life employment histories, in terms of employment sequences with annual information on the employment situation for each year of age between 50 and 70 (21 time points). Two examples of employment sequences are presented in Supplementary Table S1.

#### Life course conditions

The key life course conditions under consideration are previous labor market participation, partnership circumstances and parenthood. Each of these factors is derived from the life history interview and assessed separately for early-adulthood (20–34 years) and mid-adulthood (35–49 years). The former period covers a phase of life in which the individual typically becomes independent, including end of full-time education and first experiences in the labor market, while the latter refers to a phase with increasing responsibilities both in the labor market and parenthood (Willis & Martin, 2005).

#### Labor market participation

We combine information on full-time and part-time work and calculate the proportion of time spent in employment (for early- and mid-adulthood separately). Part-time work, in that case, counted as 0.5 full-time work. Because a first inspection showed that values did not vary a lot (most men were continuously working), the scores are regrouped into a binary indicator of whether the respondent was working most of the time (more than 75%) or not in each time period.

#### Partnership

We calculate the proportion of years spent in a cohabiting partnership (regardless of marital status), again regrouped into a binary indicator of whether the respondent spent the majority of time in a partnership or not (more than 75%).

#### Parenthood

The life history data allows us to measure the number of children (biological and nonbiological) in the household at each year of age and how old each child was in each year. On this basis, we measure the maximum number of children aged between 0 and 16 years, again both for early adulthood and mid adulthood. We regroup this information into “no children,” “1 or 2 children,” and “3 or more children.”

#### Additional variables

We include a number of additional measures, mainly as control variables in multivariable analyses. In addition to sex and year of birth, there are two indicators of childhood adversity, one measure of occupational class, and two indicators of life course health. We regroup year of birth into three different cohorts (“born 1925 or earlier”, “born between 1926 and 1930”, and “born after 1930”). Childhood adversity is included because it may influence both adulthood conditions and late life employment histories. The first measure of childhood adversity assesses whether the respondents reported “less than 10 books” in the household at age ten (Evans, Kelley, Sikora, & Treiman, 2010). The second measures the housing quality and whether none of the following characteristics were available at home (again at age 10): fixed bath, cold running water supply, hot running water supply, inside toilet, and central heating (Dedman, Gunnell, Davey Smith, & Frankel, 2001). Both variables of childhood adversity had some missing values (6% for books and 5% for housing quality), mostly when the respondent was not living in a house at age 10 (e.g., boarding school or children’s home). In both cases, further analyses revealed that missing values were not related to employment histories, life course conditions, birth cohort, or gender. It seems, thus, unlikely that missingness affects our results and we decided not to apply imputation strategies. Occupational position is measured according to the National Statistics Socioeconomic Classification (NS-SEC) (Rose, Pevalin, & O’Reilly, 2005) using information of the last occupation (or current occupation if still employed at Wave 3). The NS-SEC is the primary social classification in the United Kingdom and our study uses the three-category version to allocate individuals’ class position (“managers and professionals”, “intermediate occupations”, and “routine and manual occupations”). Again, it is likely that occupational position confounds the association between adulthood condition and late life histories, because a favorable social position is related to continued and longer working careers (Carr et al., 2016; Wahrendorf & Siegrist, 2014). Where no information on occupational position was available (e.g., “not working”) respondents were coded with “class not known”. Life course health is measured with self-reported health during childhood on a Likert scale with the possible choices being “excellent”, “very good”, “good”, “fair”, or “poor”, and where poor health is assumed in case health is rated less than good. In 0.4% of the cases, information on childhood health was missing. In addition, an indicator measures whether the respondent ever had a physical injury that had permanent effect on daily life during his or her life course. Including these health indicators in multivariable analyses control for the reduces the effect of ill health affecting both life course conditions during adulthood and specific patterns of late life employment histories. All covariates are summarized in Table 1.

**Table 1.** Sample Description: Observations (Number) and Percentage (Col. %), *n* = 2,298

	Categories	Number	Col. %
Early adulthood (ages 20–34)			
Work participation	Not mainly working	898	39.1
	Mainly working	1,400	60.9
Partnership	Not mainly partnered	1,291	56.2
	Mainly partnered	1,007	43.8
Children	No children	526	22.9
	One or two children	1,183	51.5
	Three or more children	589	25.6
Mid adulthood (ages 35–49)			
Work participation	Not mainly working	855	37.2
	Mainly working	1,443	62.8
Partnership	Not mainly partnered	370	16.1
	Mainly partnered	1,928	83.9
Children	No children	365	15.9
	One or two children	1,142	49.7
	Three or more children	791	34.4
Sex	Male	1,103	48.0
	Female	1,195	52.0
Cohort	Born 1925 or earlier	610	26.5
	Born 1926–1930	589	25.6
	Born after 1930	1,099	47.8
Books in childhood (missing:147)	Many	1,408	65.5
	Few	743	34.5
Poor housing quality in childhood (missing:114)	Yes	180	8.2
	No	2,004	91.8
Occupational class	Manager and professionals	680	29.6
	Intermediate occupations	559	24.3
	Routine and manual occupations	952	41.4
	Class not known	107	4.7
Poor self-rated health in childhood (missing:8)	Yes	262	11.4
	No	2,028	88.6
Ever physically injured	Yes	294	12.8
	No	2,004	87.2
Total		2,298	100.0

*Note*: In case of work participation and partnership mainly refers to more than 75% of the time in the time period covered.

### Analytical Strategy

The analyses proceed in two steps. First, we apply sequence analysis and group similar late life employment histories into empirically distinct clusters (Abbott, 1995; Aisenbrey & Fasang, 2010). Second, regression models test the associations between life course factors and types of late life employment histories.

More specifically, we use sequence analyses to compare each individual’s employment history to all other histories observed in the data and calculate differences (pairwise distances) of each single sequence to another. To calculate differences, we use optimal matching (OM), which considers duration, timing and ordering when comparing sequences to one another—three key aspects to characterize life trajectories (for a comparison and performance of different distance measures, see Studer & Ritschard, 2016). In the case of OM, differences (or “distances”) are calculated based on the number of operations that are necessary to make one sequence identical to another, either by substituting states (so-called “substitution costs”) or by inserting and deleting states (so called “indel costs”). In our case, we follow the standard practice (Abbott & Tsay, 2000) and set the substitution costs to twice the indel cost (1.0 and 0.5, respectively). Comparing each sequence to all other sequences results in a matrix that quantifies the distances for each pair of individuals in the sample (i.e., a 2,298 × 2,298 matrix in our study). This matrix can then be used in cluster analyses, enabling us to identify empirically homogeneous groups with similar sequences (for an alternative approach grouping individuals on the basis of their distances to predefined “model” histories, see Worts et al., 2016). We performed partitioning around medoids clustering. To determine the most appropriate number of clusters, we compared solutions with between 6 and 12 clusters, based on commonly used measures of cluster quality: the Average silhouette width (Studer, 2013) and the within/between cluster distance ratio (WB-ratio) (Aisenbrey & Fasang, 2010). Also, we looked at the resulting cluster sizes and verified each cluster solution in terms of its content validity and whether a higher cluster solution added another cluster of interest. For the analyses, we decided to adopt an eight-cluster solution, as all solutions revealed a reasonable structure, and because this turned out to be the solution with distinct and informative clusters. Details on partition quality measures can be found in the Supplementary Appendix (Supplementary Table S2).

**Figure 1.**
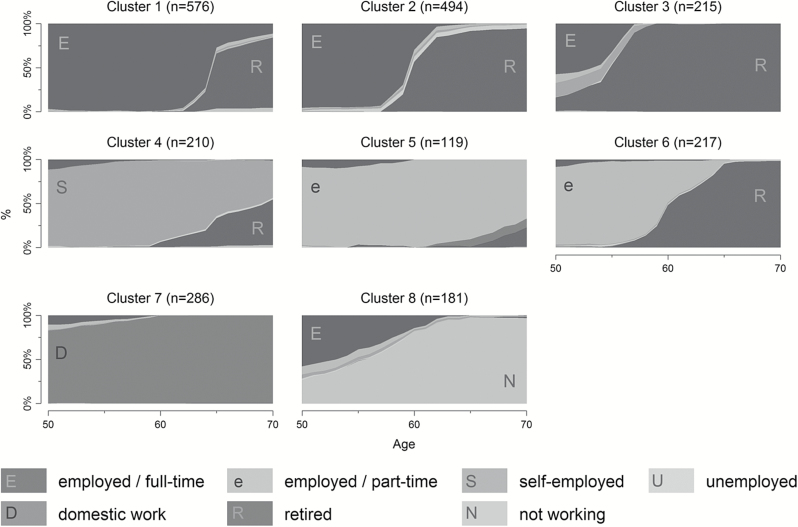
Clusters of Late Life Employment Histories. Chronograms, *n* = 2,298.

An overview of resulting clusters is given in Table 2 and Figure 1 presents chronograms of the clusters (the prevalence of each occupational situation in percent for each age). The distribution of clusters by gender is presented in Table 3, including test of significance (chi-square). Calculations and graphs are based on the SADI-package in Stata (Halpin, 2014) and the sq-Package of Brzinsky-Fay and Kohler (Brzinsky-Fay, Kohler, & Luniak, 2006). Also, we use the TraMineR package in R for calculating dissimilarities (Gabadinho, Ritschard, Muller, & Studer, 2011) and the packages WeightedCluster (Studer, 2013) and fpc (Hennig, 2015) for cluster calculation and partition quality measures respectively.

**Table 2.** Late Life Employment Clusters, Observations (Number), Percentage (Col. %), and Dominant States

	Number	Col. %	Description	Dominant states^a^	Short label
*Cluster*					
*1*	576	25.1	FT employed and retirement after age 60	FT employed: 73.6 %; Retired: 21.8%	FTE (*R* > 60)
*2*	494	21.5	FT employed and retirement mainly at age 60	FT employed: 45.8 %; Retired: 46.2%	FTE (*R* 60)
*3*	215	9.4	FT employed and retirement before age 60	FT employed: 14.9 %; Retired: 77.2%	FTE (*R* < 60)
*4*	210	9.1	Self-employed dominant	Self-employed: 79.2%; Retired: 14.6%	*SE*
*5*	119	5.2	PT employed dominant	PT employed: 88.9%; Retired: 3.6%	PT
*6*	217	9.4	PT employed and retirement mainly at age 60	PT employed: 49.9%; Retired: 45.7%	PT (*R* 60)
*7*	286	12.4	Domestic work dominant	Domestic work: 94.9%; FT employed: 3.0 %;	DW
*8*	181	7.9	Not working dominant	FT employed: 21.3 %; Not working: 70.9%	NW
*Total*	2,298	100.0			

*Note*: DW = Domestic work ; FT = Full-time; FTE = Full-time employed; NW = Not working; PT = Part-time.

^a^Cells show the two most dominant states and the percentage of time spent between ages 50 and 70

**Table 3.** Distribution of Late Life Employment Clusters by Gender, Observations (No.), and Percentage (Col. %)

		Women		Men	
		Number	Col. %	Number	Col. %
Cluster	Short description				
1	FT employed and retirement after age 60	141	11.8	435	39.4
2	FT employed and retirement mainly at age 60	205	17.2	289	26.2
3	FT employed and retirement before age 60	120	10.0	95	8.6
4	Self-employed dominant	64	5.4	146	13.2
5	PT employed dominant	94	7.9	25	2.3
6	PT employed and retirement mainly at age 60	210	17.6	7	0.6
7	Domestic work dominant	277	23.2	9	0.8
8	Not working dominant	84	7.0	97	8.8
Total		1,103	100.0	1,195	100.0
		(Chi^2^(7) = 678.65; *p* < .001)			

The second set of analyses examines associations between life course conditions and clusters of late life employment histories. For this, Table 4 presents the distribution of cluster membership for each studied life course condition (i.e., labor market participation, partnership, and parenthood). Thereafter, we formally test associations for men and women separately based on multivariable multinomial regression models with cluster membership as the dependent variable (Tables 5 and 6). Models are calculated for each life course condition separately and are adjusted for birth cohort, number of books, housing quality, self-rated health during childhood, life course major disability, and occupational position. To facilitate the presentation and interpretation of findings, we follow recent recommendations and present average marginal effects (denoted as “AME”) together with levels of significance (Williams, 2012). AME are more intuitive and easier to interpret, compared to odds ratios, and we do not need to use one cluster as a reference cluster to interpret the results. AME are presented in percent. For example, if the AME is 5.0% for cluster 1 for those who were mainly partnered in early adulthood, this means that the probability of being part of the cluster is on average 5% points higher compared with those who were not mainly partnered. Finally, besides calculating models for men and women separately, we also formally test if relationships between each life course condition and cluster differ between men and women. To do so, we combine data of men and women and test interactions between sex and life course conditions. Results are presented in Supplementary Table S3.

**Table 4.** Distribution of Late Life Employment Clusters by Early- and Mid-adulthood Conditions, Percentage (Col. %)

		Not mainly working	Mainly working	Not mainly partnered	Mainly partnered	No children	One or two children	Two or more children
		Col. %	Col. %	Col. %	Col. %	Col. %	Col. %	Col. %
		Early adulthood (ages 20–34)						
Cluster								
1	FT employed and retirement after age 60	10.8	34.2	28.7	20.4	29.7	24.9	21.4
2	FT employed and retirement mainly at age 60	14.0	26.3	22.4	20.4	25.3	19.9	21.2
3	FT employed and retirement before age 60	9.0	9.6	9.5	9.2	8.7	10.1	8.5
4	Self-employed dominant	5.8	11.3	10.1	7.8	8.7	9.6	8.7
5	PT employed dominant	10.7	1.6	3.7	7.1	4.6	5.4	5.3
6	PT employed and retirement mainly at age 60	20.0	2.6	6.7	12.9	4.8	10.7	11.0
7	Domestic work dominant	23.4	5.4	11.4	13.8	12.2	12.3	13.1
8	Not working dominant	6.2	8.9	7.4	8.4	6.1	7.2	10.9
Total		100.0	100.0	100.0	100.0	100.0	100.0	100.0
		(Chi^2^ (7) = 584.81; *p* < .001)		(Chi^2^ (7) = 58.57; *p* < .001)		(Chi^2^ (14) = 40.48; *p* < .001)		
		Mid adulthood (ages 35–49)						
Cluster								
1	FT employed and retirement after age 60	6.4	36.1	26.8	24.7	27.1	24.4	25.0
2	FT employed and retirement mainly at age 60	9.6	28.6	23.2	21.2	26.8	20.7	20.2
3	FT employed and retirement before age 60	7.6	10.4	10.0	9.2	10.4	9.8	8.2
4	Self-employed dominant	5.1	11.5	9.2	9.1	8.2	9.3	9.4
5	PT employed dominant	12.0	1.1	4.6	5.3	4.1	5.7	4.9
6	PT employed and retirement mainly at age 60	22.2	1.9	5.1	10.3	3.8	10.6	10.4
7	Domestic work dominant	29.6	2.3	10.8	12.8	12.6	12.2	12.8
8	Not working dominant	7.4	8.2	10.3	7.4	6.8	7.4	9.1
Total		100.0	100.0	100.0	100.0	100.0	100.0	100.0
		(Chi^2^ (7) = 988.16; *p* < .001)		(Chi^2^ (7) = 14.44; *p* = .044)		(Chi^2^ (14) = 27.30; *p* = .018)		

*Note*: Sequences are characterized in Table 2 and Figure 1.

**Table 5.** Associations Between Work, Partnership and Children Histories (in Early and Mid-adulthood), and Clusters of Late Life Employment History for Men

Short label		Cluster 1	Cluster 2	Cluster 3	Cluster 4	Cluster 5	Cluster 6	Cluster 7	Cluster 8
		FTE (*R* > 60)	FTE (*R* 60)	FTE (*R* < 60)	*SE*	PT	PT (*R* 60)	DW	NW
Early adulthood/work	Not mainly working (ref.)	0.0	0.0	0.0	0.0	0.0	0.0	0.0	0.0
	Mainly working	17.8^***^	17.1^***^	−3.5	−4.0	−26.2^***^	−3.7	−1.1	3.6
Early adulthood/partnership	Not mainly partnered (ref.)	0.0	0.0	0.0	0.0	0.0	0.0	0.0	0.0
	Mainly partnered	−1.6	0.9	−1.4	0.5	1.4	−0.6	0.2	0.7
Early adulthood/children	No children (ref.)	0.0	0.0	0.0	0.0	0.0	0.0	0.0	0.0
	One or two children	−7.3^*^	−1.2	6.1^***^	2.3	0.6	−0.8	−1.0	1.3
	Three or more children	−14.1^***^	2.2	4.1	3.2	0.1	−1.0	−0.3	5.8^*^
Mid adulthood/work	Not mainly working (ref.)	0.0	0.0	0.0	0.0	0.0	0.0	0.0	0.0
	Mainly working	36.9^***^	23.1^***^	−4.2	−5.8	−31.5^***^	−3.8	−3.0	−11.6^*^
Mid adulthood/partnership	Not mainly partnered (ref.)	0.0	0.0	0.0	0.0	0.0	0.0	0.0	0.0
	Mainly partnered	−4.0	1.2	2.8	−0.5	1.8^*^	−0.1	−0.5	−0.7
Mid adulthood/children	No children (ref.)	0.0	0.0	0.0	0.0	0.0	0.0	0.0	0.0
	One or two children	−6.7	−2.6	5.5^*^	1.6	1.4	0.1	−1.3	2.0
	Three or more children	−6.6	−2.7	2.6	3.7	0.6	−0.5	−0.5	3.5

*Note*: Average marginal effects and levels of significance based on multinomial regression analysis, *n* = 1,024. All estimates are calculated in separate models and are adjusted for cohort, number of books, housing quality, self-rated health during childhood, life course major disability and occupational position. Sequences are characterized in Table 2 and Figure 1.

*p < .05. **p < .01. ***p < .001.

**Table 6.** Associations Between Work, Partnership and Children History (in Early and Mid-adulthood), and Clusters of Late Life Employment History for Women

		Cluster 1	Cluster 2	Cluster 3	Cluster 4	Cluster 5	Cluster 6	Cluster 7	Cluster 8
		FTE (*R* > 60)	FTE (*R* 60)	FTE (*R* < 60)	*SE*	PT	PT (*R* 60)	DW	NW
Early adulthood/work	Not mainly working (ref.)	0.0	0.0	0.0	0.0	0.0	0.0	0.0	0.0
	Mainly working	5.8^*^	8.6^***^	3.8	2.3	−3.8^*^	−10.4^***^	−7.6^**^	1.4
Early adulthood/ partnership	Not mainly partnered (ref.)	0.0	0.0	0.0	0.0	0.0	0.0	0.0	0.0
	Mainly partnered	0.8	−0.4	0.1	0.6	1.7	2.0	−4.7	0.0
Early adulthood/children	No children (ref.)	0.0	0.0	0.0	0.0	0.0	0.0	0.0	0.0
	One or two children	1.9	−8.1^*^	−5.7^*^	1.2	0.6	8.6^**^	−0.1	1.6
	Three or more children	3.7	−6.8	−7.4^*^	−0.7	−0.4	6.2^*^	2.1	3.4
Mid adulthood/work	Not mainly working (ref.)	0.0	0.0	0.0	0.0	0.0	0.0	0.0	0.0
	Mainly working	15.1^***^	20.6^***^	6.6^**^	4.7^**^	−6.4^***^	−16.4^***^	−24.9^***^	0.8
Mid adulthood/partnership	Not mainly partnered (ref.)	0.0	0.0	0.0	0.0	0.0	0.0	0.0	0.0
	Mainly partnered	−3.5	−8.3^*^	−3.7	0.6	−0.1	10.0^***^	7.3^*^	−2.3
Mid adulthood/children	No children (ref.)	0.0	0.0	0.0	0.0	0.0	0.0	0.0	0.0
	One or two children	0.7	−10.6^**^	−8.8^**^	1.4	2.2	11.5^***^	3.5	0.1
	Three or more children	2.2	−10.7^**^	−9.8^**^	−0.9	0.8	10.2^***^	6.1	2.0

*Note*: Average marginal effects as per cent and levels of significance based on multinomial regression analysis, *n* = 1,117. All estimates are calculated in separate models and are adjusted for cohort, number of books, housing quality, self-rated health during childhood, life course major disability and occupational position. Sequences are characterized in Table 2 and Figure 1.

*p < .05. **p < .01. ***p < .001.

## Results

### Sample Description

The total sample includes slightly more women than men (1,195 vs. 1,103). Most people were born after 1930, with a mean age of 78 years when answering the life history interview (not shown in the table). As regards life course conditions, the majority were working most of the time during early and mid-adulthood (61% and 63%, respectively), and most respondents had children aged 0–16 years in both periods as well. While slightly less than half of the persons reported that they were mainly partnered in early adulthood, values are clearly higher for mid-adulthood (˃80%; for details, see Table 1).

### Types of Late Life Employment Histories

Which types of late life employment histories can be distinguished in the sample? To answer this question (first research question), Table 2 and Figure 1 describe eight different clusters and Table 3 investigates their distribution by gender. Clusters 1–3 are dominated by histories of full-time employment with varying age of retirement. The majority of the total sample (56%) belongs to one of these three clusters. Cluster 4, in contrast, is dominated by persons who were self-employed and either entered retirement later than in the first three clusters or not at all (until age 70). Clusters 5 and 6 capture part-time employees (with or without retirement). Cluster 7 is dominated by domestic work, and cluster 8 includes those who were not working most of the time for any other reason. Table 3 clearly shows that cluster membership varies significantly by sex (*p* < .001). Most men belong to cluster 1 (employed full-time and entering retirement after 60) and in only a few cases men worked part-time in later life. Women, in contrast, often belong to cluster 7 (domestic work) or clusters with retirement at age 60 (either preceded by full or part-time work).

### Associations Between Life Course Conditions and Late Life Employment Histories

Table 4 presents bivariate associations for each life course condition and type of late life employment histories. Those who mainly worked in early or mid-adulthood are also more likely to be part of clusters that are dominated by full-time employment or self-employment (clusters 1, 2, or 4). For example, while 34% of those who mainly worked during early adulthood are part of cluster 1 (FT-work with late retirement), values are about 23% points lower (11%) for those who were not mainly working in early adulthood. These associations are additionally studies separately for men and women in multinomial regressions. Due to the large number of parameters involved and space limitations, Tables 5 and 6 only presents estimates of the fully adjusted model for each life condition (tested separately). These models adjust for cohort membership, childhood adversity, self-rated health during childhood, life course major disability, and occupational position. Models were also compared to nested models (e.g., excluding occupational position) and estimates remain stable. Furthermore, results testing interactions between sex and life conditions upon cluster membership are presented in Supplementary Table S3. In sum, three findings should be noted:

First, we again see a clear positive association between previous labor market participation and continued full-time employment in later life. This is particularly true for those who mainly worked during mid-adulthood and is more pronounced for men than for women (*p* value for interactions < .001). Likewise, we see that paid work in adulthood (“mainly working”) is negatively associated with part-time employment histories at ages 50–70 (cluster 5), suggesting that people who work part-time in adulthood tend to continue working part-time in later life, rather than reducing their working hours.

A second finding worth noting refers to gender differences in the associations between parenthood and cluster membership: For men, a higher number of children during early adulthood is related to a significantly lower probability of continued full-time employment with retirement at age 65 or later (cluster 1). For women, in contrast, no such association is found, and rather, we observe that retirement before or at age 60 (clusters 2 and 3, respectively) is less likely for those with a high number of children. Again, interactions are statistically significant (*p* < .001, see Supplementary Table S3 for details). Also, it is important to note that we found no association between number of children and histories of domestic work.

Third, turning to partnership, women who were mainly partnered during mid-adulthood appear more likely to be part of cluster 6 (part-time work with retirement around age 60) and of cluster 7 (domestic work), compared to women who were mainly not partnered. We do not observe this association for men.

## Discussion

This contribution uses life history data from ELSA with detailed information on late life employment histories for men and women in England. As a first aim, we summarized employment histories based on sequence analysis, and second, we investigated associations between work, partnership and parenthood during early (ages 20–34) and mid-adulthood (ages 35–49), and types of histories. These two aims address important principles of life course research, first, to adopt a more “holistic” perspective that describes entire employment histories of later life (based on sequence analyses) (Aisenbrey & Fasang, 2010; Sackmann & Wingens, 2003), and second to consider conditions at earlier stages of the life course to explain late life employment histories (Dannefer, 2003; Elder et al., 2003).

Regarding the first aim, we found eight different types of histories that mainly varied by labor market status (full-time, part-time, or self-employed) and age of retirement. The majority of histories were dominated by full-time employees with retirement before, at, or after age 60. Most men belonged to one of these histories and, in particular, to histories with retirement after age 60. This is consistent with our expectations from the Introduction. Interestingly, we also found a cluster that is dominated by self-employment with comparatively later retirement. This may be because private pension levels for the self-employed are lower than for employees in our sample, resulting in increased incentives (and necessity) to work longer. Remaining clusters consisted of histories of part-time work (retirement around age 60 or no retirement), continuous domestic work, or other forms of nonemployment. Again, consistent with our expectations, women were more likely to have histories marked by part-time work or domestic work, possibly due to traditional gender roles. In sum, our findings illustrate the variety of late life employment histories and the benefit of a comprehensive assessment that is not limited to a single aspect, such as timing of retirement. This, on the one hand, made clear that retirement timing is interlocked with employment status, whereby self-employed workers tend to work longer compared to employed workers (Cahill et al., 2012; Wahrendorf, Akinwale, Landy, Matthews, & Blane, 2016). On the other hand, by including all older adults—regardless of whether they retired or worked beforehand—we also identified employment histories that would have been excluded otherwise (Worts et al., 2016), such as histories of domestic work for women and those not ending in retirement. By including these histories, a fuller picture of late life employment histories was possible.

Regarding the second aim of the study, in accordance with the life course perspective, we found that previous work and family experiences are linked to later patterns of employment histories, especially labor market participation during adulthood. Specifically, for both men and women, those who had strong ties to the labor market during early and mid-adulthood tended to have longer working lives, with associations more pronounced for men. Overall, this contradicts the assumptions that people retire once they can afford to, because those who worked throughout working life (probably with continued pension contributions) work later on. Rather, it supports the idea of cumulative advantages (Dannefer, 2003), where those who established solid ties to the labor market at an earlier stage of their life course are also more likely to have stable and continued histories later on. Findings for partnership and parenthood were less apparent but again pointed to interesting gender differences. While a high number of children tended to lead to longer working lives for men, the opposite was the case for women. With regards to partnership, we only found an association for women (but not for men), such that partnered women were more likely to have histories of domestic work or part-time work. This contradicts previous studies where a high number of children were linked to an extended working life for women (Finch, 2014) and it points to traditional gender roles in the division of paid and unpaid work within partnerships.

More generally, our results highlight the importance of a life course perspective. Particularly, we see that individuals construct their lives within a complex set of opportunities and constraints, defined in part by individual experiences and by interdependencies between different domains of life, together with national policies, historical and cultural contexts. These factors determine how people participate in the labor market at older ages, as well as how life course conditions are linked to types of late life employment histories. As such, the life course perspective offered a comprehensive framework helping to elucidate labor market involvement at older ages.

Our study has several strengths, including a large study sample, detailed life history data, the use of sequence analyses to derive empirically distinct clusters, and the inclusion of several covariates. When discussing our findings we must, however, consider several limitations.

First, the core measures of our study were collected retrospectively, namely life course conditions and employment histories between age 50 and 70 years. As such, respondents may have remembered information inaccurately, or remembered details rosier than they were. We thus need to consider a potential recall bias. Yet, there is increasing support that retrospective data (in particular those collected via “calendar interviews” as the case in ELSA) provide reliable and valid information (e.g., Belli, 1998).

Second, our study is limited to one country, and therefore, far reaching conclusions on contextual influences require future analyses with more countries that allow the comparison of different contexts. For example, given that pension schemes in England are largely organized on a private basis, and that levels of public pensions are generally lower compared to other European countries (Börsch-Supan, Brugiavini, & Croda, 2009), findings may be different in other countries. The link between stronger ties to the labor market during adulthood and extended working, for example, may be different in countries with more generous pension schemes, because of entitled benefits (Worts et al., 2016). For the English context, though, our study clearly shows that previous labor market involvement matters for late life employment outcomes. In a similar vein, our study would benefit from additional information about specific aspects, such as individual memberships of pension schemes of the respondents, reasons for retiring or for working beyond the state pension age or both. However, this information is not available in the ELSA life history data. Also, to investigate these questions in more detail would require larger sample sizes allowing meaningful analyses of subgroups.

Third, in our study, employment sequences were measured annually and employment spells were recorded in the interview if they were longer than 6 months. As a consequence, we may have bypassed some short spells and underestimated the diversity of employment sequences. Likewise, some respondents were excluded because they had incomplete employment histories (missing state-information within sequences). Hereby, it is possible that we excluded incomplete histories with particular patterns. Yet, additional analyses revealed that histories were complete for 82% of all people aged 70 or older. Also, we found no support that the incomplete sequences may have affected the identified clusters but rather led to different cluster sizes only. It is also unlikely that our strategy may have affected the associations between life course conditions and later employment histories. Therefore, we decided not to apply procedures of imputing incomplete sequences (Halpin, 2015). Furthermore, although we distinguished seven different occupational situations in our study, future studies may go further and include additional information when defining occupational states (e.g., a more differentiated measure of those not working, including information on voluntary work). This may help to explore under which circumstances people are more likely to participate in voluntary work at older ages. This information, however, is not available in the ELSA life history data, and again, we need to question whether the existing samples size is large enough to warrant the additional complexity that would have been involved in our study.

Fourth, we found that women were more likely than men to be part of specific types of late life employment histories (e.g., domestic histories) and thereby confirmed findings from other studies (Worts et al., 2016). While it is arguable that the clustering of histories should have been conducted for men and women separately, this procedure would have two consequences for our study: First, it would prevent us from studying gender differences in the likelihood of belonging to the same types of histories (but only allow to see if both sexes come up with similar histories). Second, our regression analyses would have had to deal with different outcomes for men and women (different cluster solutions), making comparisons between sexes complicated (and test of interactions impossible).

Finally, we have to ask how our results align with changing workforces in Europe. Our results rely on a sample of men and women born in 1937 or earlier. They grew up under specific circumstances (e.g., 1930s depression) and had their late life employment histories during a specific historic period (mostly between 1987 and 2007). Therefore, although unavoidable for methodological reasons, the relevance of our results for today’s workforce is possibly different. In fact, given that the nature of work and employment has changed significantly over the past few decades (often combined with instability and discontinuity of employment histories [Gallie 2013; Kalleberg 2012]), discontinuous histories may be more common today than in our sample. Nevertheless, employment trajectories of previous cohorts may be informative in predicting employment behaviors of future cohorts.

In sum, this study distinguishes different types of employment history and shows that the probability of belonging to specific types of history is related to previous life course conditions. Based on our findings, a first implication is that policies aiming at increasing the proportion of older workers should not only address later stages of the life course but also early and mid-adulthood. A second, rather theoretical implication is that our study provides a good example on how the life course perspective helps to conceptualize and elucidate employment patterns at older ages. In that respect, the focus of our analyses was on studying entire patterns of late life employment histories and on ways in which work family formation during adulthood have long-lasting consequences for later employment patterns. But we also found clear support that historical and cultural contexts shape histories as well. Finally, a rather methodological conclusion is that our study illustrates the value of retrospective life history data in analyzing determinants of extended working lives.

## Supplementary Material

Supplementary data are available at *The Journals of Gerontology, Series B: Psychological Sciences and Social Sciences* online.

## Funding

The funding is provided by the National Institute of Aging in the United States, and a consortium of U.K. government departments co-ordinated by the Office for National Statistics. The developers and funders of ELSA and the Archive do not bear any responsibility for the analyses or interpretations presented here. MW and HH were supported by funding from the German Research Foundation (Deutsche Forschungsgemeinschaft), project number: WA 3065/3-1. PZ, JH, and EC were funded by the Economic and Social Research Council and the Medical Research Council as part of the Lifelong Health and Well-Being (LLHW) initiative (grant number ES/L002892/1).

## Conflict of Interest

The authors declare no conflict of interest.

## Supplementary Material

Supplementary_tables_final_jgss_revision
