# Supplementary material for: Late Life Employment Histories and Their Association With Work and Family Formation During Adulthood: A Sequence Analysis Based on ELSA
Source: J Gerontol B Psychol Sci Soc Sci. 2017 May 31;73(7):1263–77. doi: 10.1093/geronb/gbx066 (PMC6146763; doi:10.1093/geronb/gbx066)
Supplement: Supplementary_tables_final_jgss_revision [file gbx066_suppl_supplementary_tables_final_jgss_revision.docx]

**Supplementary tables**

Table S1. Example of employment sequences between ages 50 and 70.

|  |  |  |
| --- | --- | --- |
| Sequence 1 | EEEEEEEEEERRRRRRRRRRR |  |
| Sequence 2 | eeeeeeUSSSSSSSRRRRRRR |  |
|  |  |  |

Note. "E"= employed full-time; "e"= employed part-time;
"U"= unemployed; "R"= retired; "S"= self-employed

Table S2. Cluster Solutions of late life employment histories.

|  |  |  |  |  |  |  |
| --- | --- | --- | --- | --- | --- | --- |
| *Number of*  *clusters* |  | Mean within  distances | Mean between  distances | WB  Ratio | ASW | Observations in  smallest cluster |
|  |  |  |  |  |  |  |
| *6* |  | 4.88 | 16.34 | 0.30 | 0.52 | 181 |
| *7* |  | 4.18 | 15.95 | 0.26 | 0.52 | 181 |
| *8* |  | 3.86 | 15.88 | 0.24 | 0.53 | 119 |
| *9* |  | 3.41 | 15.61 | 0.22 | 0.53 | 119 |
| *10* |  | 3.19 | 15.56 | 0.21 | 0.53 | 106 |
| *11* |  | 2.74 | 15.56 | 0.18 | 0.56 | 36 |
| *12* |  | 2.55 | 15.53 | 0.16 | 0.56 | 36 |
|  |  |  |  |  |  |  |
|  |  |  |  |  |  |  |

Table S3. Interactions between life course conditions and sex on clusters of late life employment history. Results of tests of significance based on multinomial regressions analysis : chi² value, degrees of freedom (df) and p-values.

|  |  | Chi² | (df) | p-value |
| --- | --- | --- | --- | --- |
|  |  |  |  |  |
| *Early adulthood / Work* | Not mainly working (ref.) |  |  |  |
|  | Mainly working * sex | 38.10 | (7) | < 0.001 |
|  |  |  |  |  |
| *Early adulthood / Partnership* | Not mainly partnered (ref.) |  |  |  |
|  | Mainly partnered * sex | 6.00 | (7) | 0.540 |
|  |  |  |  |  |
| *Early adulthood / Children* | No children (ref.) |  |  |  |
|  | 1 or 2 children * sex | 22.43 | (7) | 0.002 |
|  | 3 or more children * sex | 24.09 | (7) | 0.001 |
|  |  |  |  |  |
|  |  |  |  |  |
| *Mid adulthood / Work* | Not mainly working (ref.) |  |  |  |
|  | Mainly working * sex | 96.48 | (7) | < 0.001 |
|  |  |  |  |  |
| *Mid adulthood / Partnership* | Not mainly partnered (ref.) |  |  |  |
|  | Mainly partnered * sex | 25.91 | (7) | < 0.001 |
|  |  |  |  |  |
| *Mid adulthood / Children* | No children (ref.) |  |  |  |
|  | 1 or 2 children * sex | 28.73 | (7) | < 0.001 |
|  | 3 or more children * sex | 25.80 | (7) | < 0.001 |
|  |  |  |  |  |

Note. Models are calculated separately for each life course conditions based on the total sample. Values refer
to the joint test that all respective life course condition-sex interactions are zero.
